# German Medical Science – Journal for Medical Education: Respectable and very much in motion

**DOI:** 10.3205/zma000954

**Published:** 2015-02-11

**Authors:** Götz Fabry, Martin R. Fischer

**Affiliations:** 1 Albert-Ludwig-Universität Freiburg, Abt. für Med. Psychologie, Freiburg/Breisgau, Deutschland; 2 GMS Zeitschrift für Medizinische Ausbildung, stellv. Schriftleiter, Erlangen, Deutschland; 3 Klinikum der Universität München, Institut für Didaktik und Ausbildungsforschung in der Medizin, München, Deutschland; 4 GMS Zeitschrift für Medizinische Ausbildung, Schriftleiter, Erlangen, Deutschland

## Editorial

The first issue of a new volume, which is the 32^nd^ of our Journal for Medical Education, is a good opportunity to take a look on what we have achieved during the last year, and what lies ahead of us as there are some innovations coming up that document the development of our journal. First of all some data: We published 35 articles in 2014. A good half of them (16) are research articles, almost a third are project reports (11), the remaining ones are reviews (1), position papers (3), and comments (4). These numbers illustrate that our journal lives up to its self-declared mission i.e. to promote the scientific discourse within the entire domain of education and professional development in medicine, dentistry and veterinary medicine as well as in the health professions overall. However, there is still room for improvement. From an editorial perspective we would wish to have more review articles since we consider the integration of insights from individual studies of utmost importance as these become increasingly difficult to keep track of in many domains.

**Figure 1:**
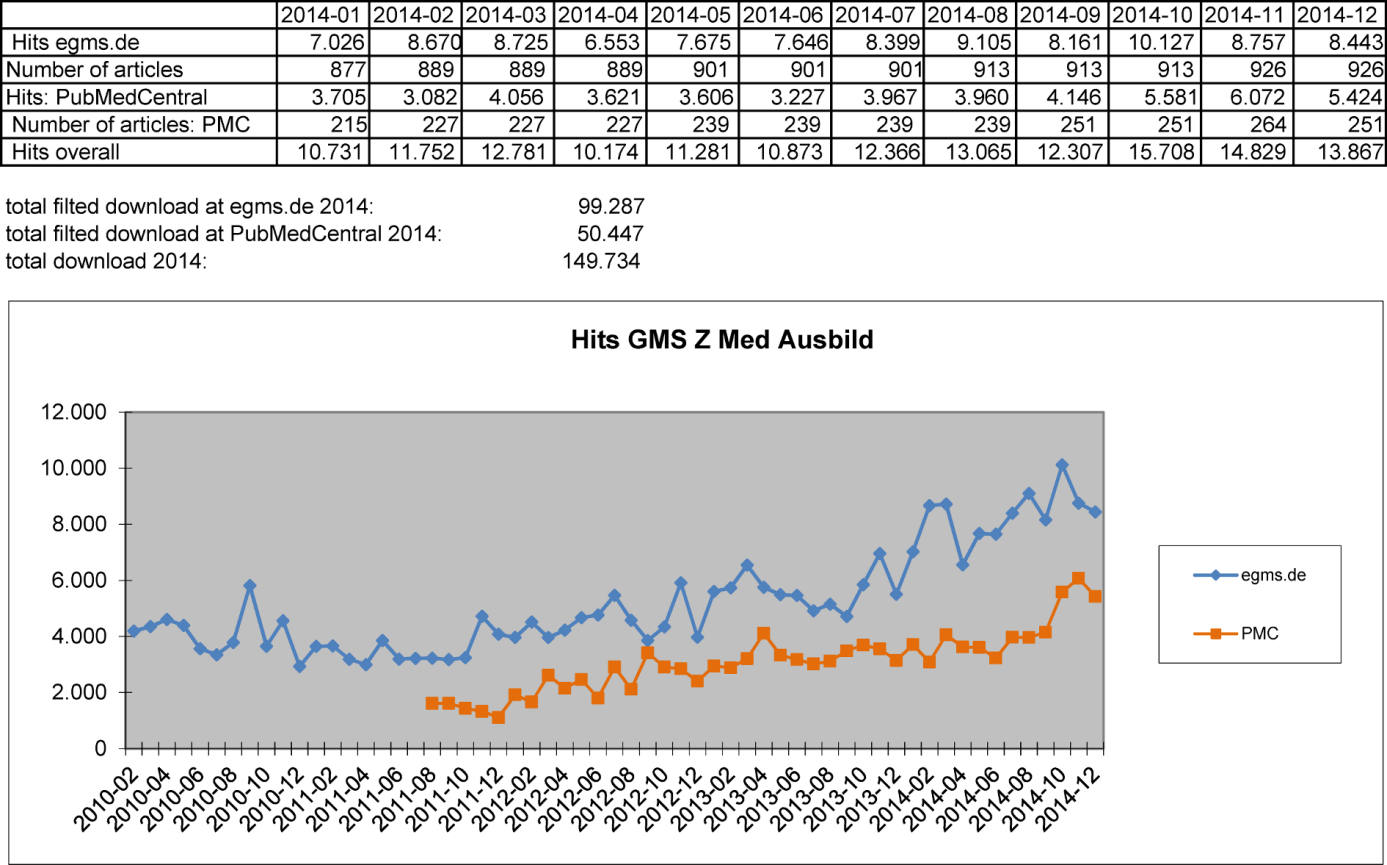
Download statistic – journal "GMS Zeitschrift für Medizinische Ausbildung"

That we are also on the right track for our readers is documented by the growing access of our sites: Both on our “home” platform GMS [http://www.egms.de/en/index.htm] and on PubMed where our articles are also available as full text we enjoy growing popularity (see Figure 1 (Fig. 1)). In 2014 we counted almost 100.000 hits on GMS. On PubMed where our journal is listed only since 2011 we still had almost 50.000 hits. Both measures are corrected for automated queries. Although we are really excited about this response we still want to develop the proliferation of our journal without making any compromises in terms of the quality of our articles. To achieve this and to come closer to the Impact Factor as an important milestone for our journal, we introduce three innovations that we hope will generate positive impetus for this development:

1. The most important change relates to our editorial board [http://www.egms.de/en/journals/zma/about.htm#editorial] that is now more international than before. We are really happy that we could win over some distinguished colleagues from different parts of the world to support us in developing our journal. Once again we would like to give an official and very warm welcome to our new editors!
2. Further changes relate to the types of articles to be published in our journal [http://www.egms.de/en/journals/zma/authors.htm#guide]. With regard to Boyer’s definition, scholarship embraces the *discovery* of new insights, the* integration* and synthesis of what is already known and also the* application* of scientific knowledge in practice [1], [2]. According to these domains of scholarship we still consider research articles and reviews but also project reports as the most important building blocks of the scientific discourse in medical education research and practice. However, to clearly distinguish especially between research articles and project reports turned out to be rather difficult at times. In addition, compared to research articles project reports have been set back by our author guidelines in terms of length which might have given the impression that they are also less important. To finally keep the number of article types manageable we decided to treat the three major article types equally with regard to formal aspects and to subsume them in one category under the heading “article”. Papers in this category will then be further categorized with regard to content. Such a classification is also used in other major medical education journals and especially suites the problems and questions in our domain.
3. The third innovation at the beginning of this year will hopefully turn out as a further incentive for authors to publish in our journal [3]. For the first time the GMA awards a price for the best paper in the Z Med Ausbild. The paper will be selected by the editorial board. The winner of the award for the best paper in 2014 will be announced in the next issue of our journal.

To reduce the time till then we recommend the article of the current issue of our journals as a very rewarding reading! And don’t forget to submit your best manuscripts to Z Med Ausbild!

## Competing interests

The authors declare that they have no competing interests.
